# Resistin Regulates Inflammation and Insulin Resistance in Humans via the Endocannabinoid System

**DOI:** 10.34133/research.0326

**Published:** 2024-04-02

**Authors:** Han-Mo Yang, Joonoh Kim, Baek-Kyung Kim, Hyun Ju Seo, Ju-Young Kim, Joo-Eun Lee, Jaewon Lee, Jihye You, Sooryeonhwa Jin, Yoo-Wook Kwon, Hyun-Duk Jang, Hyo-Soo Kim

**Affiliations:** ^1^ Department of Internal Medicine, Seoul National University Hospital, Seoul, Korea.; ^2^ National Research Laboratory for Stem Cell Niche, Seoul National University Hospital, Seoul, Korea.; ^3^ Innovative Research Institute for Cell Therapy, Seoul National University Hospital, Seoul, Korea.; ^4^ Molecular Medicine and Biopharmaceutical Sciences, Seoul National University, Seoul, 03080, Korea.

## Abstract

Resistin plays an important role in the pathophysiology of obesity-mediated insulin resistance in mice. However, the biology of resistin in humans is quite different from that in rodents. Therefore, the association between resistin and insulin resistance remains unclear in humans. Here, we tested whether and how the endocannabinoid system (ECS) control circulating peripheral blood mononuclear cells (PBMCs) that produce resistin and infiltrate into the adipose tissue, heart, skeletal muscle, and liver, resulting in inflammation and insulin resistance. Using human PBMCs, we investigate whether the ECS is connected to human resistin. To test whether the ECS regulates inflammation and insulin resistance in vivo, we used 2 animal models such as “humanized” nonobese diabetic/Shi-severe combined immunodeficient interleukin-2Rγ (null) (NOG) mice and “humanized” resistin mouse models, which mimic human body. In human atheromatous plaques, cannabinoid 1 receptor (CB1R)-positive macrophage was colocalized with the resistin expression. In addition, resistin was exclusively expressed in the sorted CB1R-positive cells from human PBMCs. In CB1R-positive cells, endocannabinoid ligands induced resistin expression via the p38–Sp1 pathway. In both mouse models, a high-fat diet increased the accumulation of endocannabinoid ligands in adipose tissue, which recruited the CB1R-positive cells that secrete resistin, leading to adipose tissue inflammation and insulin resistance. This phenomenon was suppressed by CB1R blockade or in resistin knockout mice. Interestingly, this process was accompanied by mitochondrial change that was induced by resistin treatment. These results provide important insights into the ECS–resistin axis, leading to the development of metabolic diseases. Therefore, the regulation of resistin via the CB1R could be a potential therapeutic strategy for cardiometabolic diseases.

## Introduction

Obesity is recognized as a potent contributor to the development of type 2 diabetes mellitus, cardiovascular diseases, and other metabolic diseases [1]. Under such conditions, obesity is known to induce chronic inflammation and insulin resistance in various organs such as the adipose tissue, skeletal muscle, liver, and the intestines [2,3]. The endocannabinoid system (ECS) has drawn attention as a potent therapeutic target for overcoming obesity because the ECS regulates energy balance, food intake, and lipid and glucose metabolism [4,5]. The ECS consists of 2 membrane receptors, cannabinoid 1 and 2 (CB1 and CB2) receptors, and their endogenous ligands, endocannabinoids, including anandamide and 2-arachidonoylglycerol (2-AG) [6].

Resistin, a 12.5-kDa cysteine-rich polypeptide, was discovered in rodents as an adipose-tissue-specific secreted protein that is down-regulated by antidiabetic thiazolidinediones [7,8]. However, human resistin is mainly produced from peripheral blood mononuclear cells (PBMCs) [9–11]. Although resistin has been implicated as a link between obesity and insulin resistance in rodents, there has been much controversy regarding the role of resistin in inducing insulin resistance in humans [8,12–14].

In this study, we searched for a mediator that could explain the differences between mice and humans in the role of resistin to induce insulin resistance. We hypothesized that there must be some relationship between the ECS and resistin, given that both systems are linked to the development of inflammation and insulin resistance. Therefore, we investigated whether this shared mediator could induce inflammation and insulin resistance in vitro and in vivo.

## Results

### The ECS is associated with resistin in human PBMCs

**Fig. 1.**
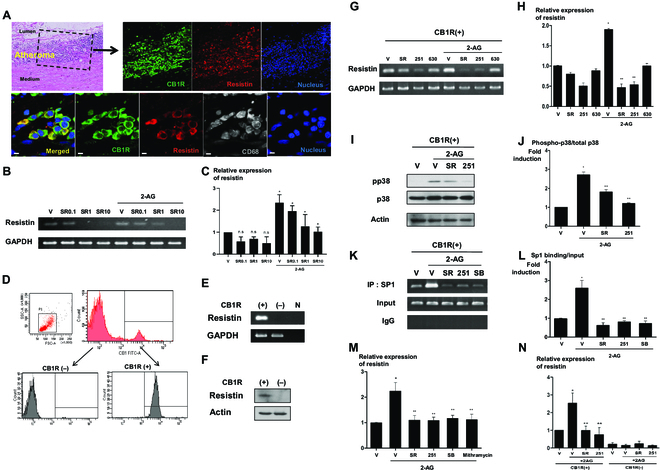
The ECS regulates resistin expression in CB1R-positive PBMCs. (A) CB1R and resistin expression in human arteries. Immunofluorescence for CB1R and resistin in human atheromatous artery. Green, CB1R; red, resistin; gray, CD68; sytox blue, nuclei. Scale bars, 10 μm. (B and C) Resistin expression in human PBMCs. Human PBMCs were treated with 10 μM 2-AG, a ligand of the ECS, with (or without) 0.1, 1, and 10 μM SR141716, a CB1R antagonist. Cells were harvested, and real-time PCR was performed for resistin (*P < 0.01, vehicle versus 2-AG, 2-AG + V versus 2-AG + SR141716; *n* = 3; hereafter, *n* represents the number of biological replicates). n.s., not significant; V, vehicle; SR, SR141716. (D to F) CB1R-positive cell sorting and resistin expression in CB1R-positve cells. (D) FACS for CB1-positive cells and purities of the isolated cells confirmed by FACS analysis. FITC, fluorescein isothiocyanate. SSC-A, side scatter area; FSC-A, foward scatter area. Resistin expression in sorted CB1R-positive cells, as determined by real-time PCR (E) and Western blot assay (F). N indicates the negative control. (G and H) Resistin expressions following treatment with 10 μM 2-AG with or without 1 μM SR141716 and 10 μM AM251 (a CB1R antagonist) in CB1R-positive cells, as determined by real-time PCR. AM630, a CB2R antagonist (*P < 0.01, vehicle versus 2-AG; **P < 0.01, 2-AG + V versus 2-AG + SR141716 or AM251; *n* = 4). 251, AM251; 630, AM630. (I and J) Western blot analysis of p38 phosphorylation in CB1R-positive cells treated with 10 μM 2-AG, 1 μM SR141716, and 10 μM AM251. (*P < 0.01, vehicle versus 2-AG; **P < 0.01, 2-AG + V versus 2-AG + SR141716 or AM251; *n* = 4). pp38, phospho-p38. (K and L) ChIP assay for the resistin promoter showed that the increased expression of resistin induced by 2-AG was induced by the enhanced binding of Sp1 on the promoter region of resistin. The increased binding activity of Sp1 induced by 2-AG was reversed by treatment with 1 μM SR141716, 10 μM AM251, and 5 μM SB203580 (a p38 inhibitor) (*P < 0.01, vehicle versus 2-AG; **P < 0.01, 2-AG + V versus 2-AG + SR141716, AM251 or SB203580; *n* = 4). SB, SB203580; IgG, immunoglobulin G. (M) Real-time PCR for resistin in CB1R-positive cells treated with 10 μM 2-AG, 1 μM SR141716, 10 μM AM251, 5 μM SB203580, and mithramycin. Moreover, mithramycin, a Sp1 inhibitor, reversed the increased expression of resistin induced by 2-AG treatment. (*P < 0.01, vehicle versus 2-AG; **P < 0.01, 2-AG + V versus 2-AG + SR141716, AM251, SB203580, or mithramycin; *n* = 4). (N) Real-time PCR for resistin in CB1R-negative and -positive cells treated with 10 μM 2-AG, 1 μM SR141716, 10 μM AM251, and 5 μM SB203580. Resistin expression was affected only in CB1R-positive fraction (*P < 0.01, vehicle versus 2-AG; **P < 0.01, 2-AG + V versus 2-AG + SR141716, AM251 or SB203580; *n* = 3).

It is known that not only the ECS but also resistin is associated with the process of atherosclerosis [4,10]. Moreover, resistin is known to be expressed in human monocytes or macrophages [12]. To investigate whether the ECS is connected to resistin, we evaluated resistin and CB1 receptor (CB1R) expression in human atheroma. Interestingly, we found cells expressing both CB1R and resistin in the CD68-positive area of the atheromatous plaque of aortas obtained from patients with aortic aneurysm (Fig. 1A). Next, we examined PBMCs that could be precursor of the infiltrated macrophages in the atheromatous plaque. After confirming resistin expression in human PBMCs, we investigated the effect of the ECS on resistin expression in PBMCs. Resistin levels were up-regulated by 2-AG, a ligand of the ECS, and down-regulated by SR141716, a CB1R antagonist, in a dose-dependent manner (Fig. 1B and C).

### Resistin is expressed only in CB1R-positive fraction of human monocytes through the 2-AG–p38–Sp1 pathway

When we analyzed whole monocytes regarding the expression of CB1R, we obtained 2 discrete populations of CB1R-positive and -negative fractions in fluorescence-activated cell sorting (FACS) (Fig. 1D). Interestingly, resistin was expressed only in CB1R-positive cells, whereas it was undetectable in the CB1R-negative fraction (Fig. 1E and F). Notably, the expression of adiponectin, one of good adipokines, was exactly opposite of that of resistin; adiponectin was expressed only in the CB1R-negative fraction while not in the CB1R-positive fraction (Fig. S1).

Additional experiments were carried out to determine whether the ECS regulates resistin expression in CB1R-positive cells. Compared to the vehicle-treated group, 2-AG treatment induced a substantial increase in resistin levels, which was reversed by CB1R antagonists such as SR141716 and AM251 but not by CB2R antagonists such as AM630 (Fig. 1G and H). Next, to understand the regulatory mechanism of resistin expression in CB1R-positive cells and the association with the ECS, we screened several pathways associated with mitogen-activated protein kinases (Fig. 1I and J and Fig. S2). Among them, the p38 pathway was under the control of the ECS, revealing that 2-AG increased the phosphorylation of p38, which was inhibited by CB1R antagonists (Fig. 1I and J). Furthermore, a chromatin immunoprecipitation assay for the resistin promoter showed that the regulation of resistin by the ECS was mediated by the increased binding of Sp1 on the promoter region of resistin. The affinity of Sp1 binding on the resistin promoter was significantly inhibited by CB1R antagonists and SB203580, a p38 inhibitor (Fig. 1K and L). Moreover, the increase in resistin levels induced by 2-AG was reversed by treatment with CB1R antagonists and a p38 inhibitor (Fig. 1M).

Last, we examined whether CB1R-negative monocytes can stimulate resistin expression by 2-AG treatment. We performed FACS and sorted CB1R-positive and -negative cells. Compared to CB1R-positive cells that showed increased expression of resistin by 2-AG treatment, CB1R-negative cells did not respond to 2-AG treatment. Both of CB1R antagonists’ treatment reversed effect of 2-AG (Fig. 1N).

Taken together, these results suggest that resistin expression in CB1R-positive cells is regulated through the p38–Sp1 pathway.

### Endocannabinoid–resistin axis regulates inflammation in vivo through the infiltration of resistin-secreting CB1R-positive cells into visceral adipose tissue

**Fig. 2.**
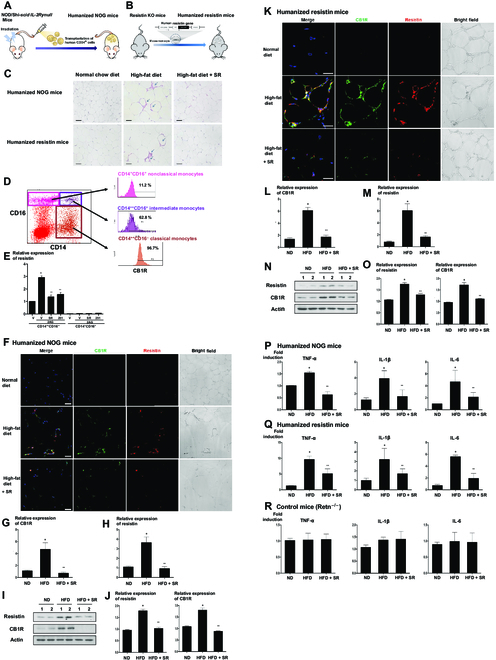
The ECS regulates inflammation in vivo by the infiltration of resistin-secreting CB1R-positive cells. (A and B) Schematic figures of 2 in vivo models. (A) To generate humanized NOG mice, the NOG mice were irradiated, and 1 × 10^5^ human CD34-positive cells were transplanted through tail vein injection. (B) Humanized resistin mice were generated after inserting human resistin gene into the monocyte of resistin knockout (KO) mice. (C) Hematoxylin and eosin staining of visceral adipose tissues. CLSs are indicated by arrows. (D) The FACS of PBMCs into several fractions according to the expression of CD14 and CD16. The CD14^++^ CD16^−^ fraction of cells, considered as classical subsets of human monocytes, was mostly CB1R-positive cells. (E) In the CD14^++^ CD16^−^ fraction of cells having CB1R, 2-AG treatment significantly increased resistin expression, which was reversed by CB1R antagonists, SR141716 or AM251 (*P < 0.01, vehicle versus 2-AG; **P < 0.01, 2-AG + V versus 2-AG + SR141716 or AM251; *n* = 4). (F to J) Visceral adipose tissues of humanized NOG mice (F) and humanized resistin mice (K). Mice were fed with a normal diet and a high-fat diet with or without intraperitoneally injected SR141716 for 8 weeks. Green, CB1R; red, resistin; 4′,6-diamidino-2-phenylindole, nuclei. Scale bars, 10 μm. Real-time PCR (G, H, L, and M) and Western blot analysis of CB1R and resistin in visceral adipose tissues of humanized NOG mice and humanized resistin mice (I, J, N, and O) (*P < 0.05, ND versus HFD; **P < 0.05, HFD versus HFD + SR; *n* = 4). ND, normal chow diet; HFD, high-fat diet. (P to R) Proinflammatory gene expressions by real-time PCR in visceral adipose tissues. TNF-α, IL-1β, and IL-6 in humanized NOG mice and humanized resistin mice, respectively (*P < 0.05, ND versus HFD; **P < 0.05, HFD versus HFD + SR; *n* = 4).

To test whether the ECS regulates inflammation and insulin resistance in vivo, we utilized 2 in vivo models: humanized nonobese diabetic/Shi-severe combined immunodeficient interleukin-2Rγ (IL-2Rγ) (null) (NOG) mice and humanized resistin mice. This was necessary because resistin expression differs in mice and humans: adipocytes in mice versus monocytes in human. The bone marrows of humanized NOG mice were replaced with human monocytes so that we could study the behavior of human monocytes in vivo with them. Humanized resistin mice were the mice that express human resistin in their monocytes after the deletion of the whole mouse resistin, resulting in physiological mimicking of human resistin (Fig. 2A and B) [15]. These mice were fed a normal chow diet or a high-fat diet with or without SR141716, which was injected intraperitoneally for 8 weeks.

Adipose tissue is known to be critical for the development of obesity-induced inflammation [16]. Therefore, we investigated the extent of the infiltrated inflammatory cells and the expression of CB1R and resistin in visceral and subcutaneous adipose tissue. Hematoxylin and eosin staining of visceral adipose tissue showed that a high-fat diet increased adipocyte size and crown-like structures (CLSs), serving as a mark of inflammation in adipose tissue. In addition, numerous cells infiltrated into the visceral adipose tissue in the high-fat diet-fed mice, compared to the normal diet-fed mice. This was reversed by treatment with SR141716 (Fig. 2C and Fig. S3A).

Next, we investigated which cells infiltrate into the visceral adipose tissue. When we divided PBMCs into several fractions according to the expression of CD14 and CD16, the CD14^++^ CD16^−^ fraction of cells were almost entirely CB1R-positive cells. CD14^++^ CD16^−^ cells are a classical subset of human monocytes that are similar to the proinflammatory M1 subsets of mouse monocytes (Fig. 2D) [16]. In the population of CD14^++^ CD16^−^ cells, resistin expression increased by treatment with 2-AG was reversed by SR141716 treatment (Fig. 2E). Since the M1 cell population of mouse monocytes is the representative cells that can infiltrate into adipose tissue under inflammatory conditions [16], we assumed that CB1R-positive cells could infiltrate into the adipose tissue under the same situation. Immunofluorescence analysis revealed the high numbers of infiltrated cells expressing both CB1R and resistin in the visceral adipose tissue of humanized NOG mice fed a high-fat diet compared to mice fed a normal chow diet. In addition, this increased infiltration of cells was reversed by treatment with the CB1R antagonist, SR141716 (Fig. 2F and Figs. S3B and S7). By contrast, such changes in visceral adipose tissue were not observed in subcutaneous adipose tissue in either mouse model (Fig. S5). We also quantified CB1R, resistin, and proinflammatory gene levels in visceral adipose tissue. The high-fat diet led to increased CB1R and resistin levels in visceral adipose tissue, which was reduced in SR141716-treated mice (Fig. 2G to J). Even in humanized resistin mice, the same results were observed (Fig. 2K to O and Fig. S3C). Moreover, we also confirmed presence of numerous cells double-positive for CB1R and even in atheromatous plaque in humanized resistin mice (Fig. S6).

The expression of proinflammatory genes such as tumor necrosis factor-α (TNF-α), IL-1β, and IL-6 increased in the visceral fat tissue of mice fed a high-fat diet. Moreover, this effect was reversed by SR141716 treatment. No significant difference was observed in the proinflammatory gene expression of resistin knockout mice (Retn^−/−^ mice) (Fig. 2P to R). These results suggest that the ECS mediates inflammation by inducing the infiltration of CB1R-positive cells that secrete resistin.

### High-fat diets increase 2-AG levels in the visceral adipose tissue, resulting in chemoattraction of CB1R-positive cells secreting resistin

**Fig. 3.**
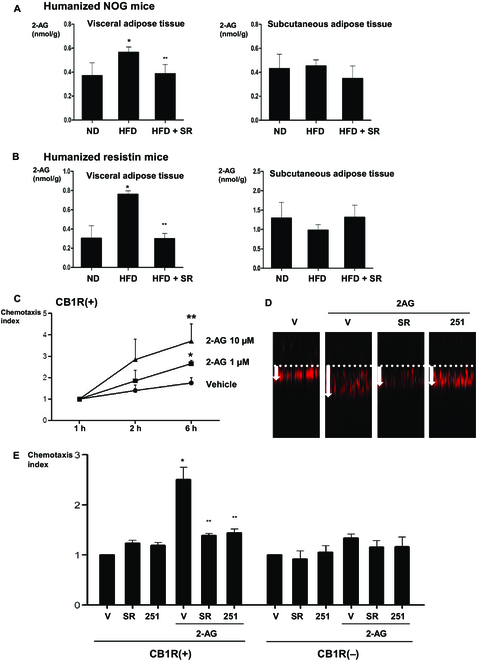
Migration of CB1R-positive cells induced by 2-AG and different 2-AG levels in adipose tissues. (A and B) 2-AG levels in visceral and subcutaneous adipose tissues of humanized NOG mice and humanized resistin mice measured by LC-MS/MS (*P < 0.05, ND versus HFD; **P < 0.05, HFD versus HFD + SR; *n* = 4). (C to E) CB1R-positive cells and CB1R-negative cells were added to the transwell insets, and 2-AG was added at 1 and 10 μM concentrations to the lower compartment. Incubation was carried out for the indicated periods of time (* *P* < 0.05, V versus 2-AG 1 μM; **P < 0.05, V versus 2-AG 10 μM; *n* = 4). (D) Representative figure of each group in the transwell migration assay. (E) Cells were pretreatment with vehicle, 1 μM SR141716, and 10 μM AM251; they were then added to the transwell inserts. The migration of the cells from the upper to lower compartment was measured (* *P* < 0.01, vehicle versus 2-AG; **P < 0.01, 2-AG + V versus 2-AG + SR141716 or AM251; *n* = 4).

To understand how a high-fat diet increases the infiltration of monocytes expressing CB1R, we hypothesized that a high-fat diet may increase the level of CB1R ligands in the visceral adipose tissue, resulting in promoting the infiltration of CB1R-positive cells. We evaluated 2-AG levels of tissues in both in vivo models by liquid chromatography-tandem mass spectrometry (LC-MS/MS). In both humanized NOG and humanized resistin mouse models, a high-fat diet increased the level of 2-AG in the visceral adipose tissue, but not in the subcutaneous adipose tissue. Furthermore, SR141716 treatment reversed the effect of the high-fat diet on 2-AG (Fig. 3A and B). We tested in vitro chemoattractant effects of 2-AG on CB1R-positive cells and confirmed the effect of 2-AG on the migration of CB1R-positive cells in a dose-dependent manner (Fig. 3C). Moreover, this chemoattraction was blocked by pretreatment with CB1R antagonists (Fig. 3D and E) and inflammatory cytokines in these cells. Furthermore, increased level of 2-AG recruited those cells into visceral adipose tissue. Regarding the role of 2-AG in human adipose tissue, it was observed that 2-AG treatment elevated resistin levels in human cell lines of stromal vascular fraction encompassing human monocytes, whereas it did not in human cell lines of white adipose tissue. This implies that the effectiveness of 2-AG is exclusive to human monocytes and does not extend to human adipocytes (Fig. S9).

### Endocannabinoid–resistin axis regulates high-fat diet-induced insulin resistance in in vivo models

**Fig. 4.**
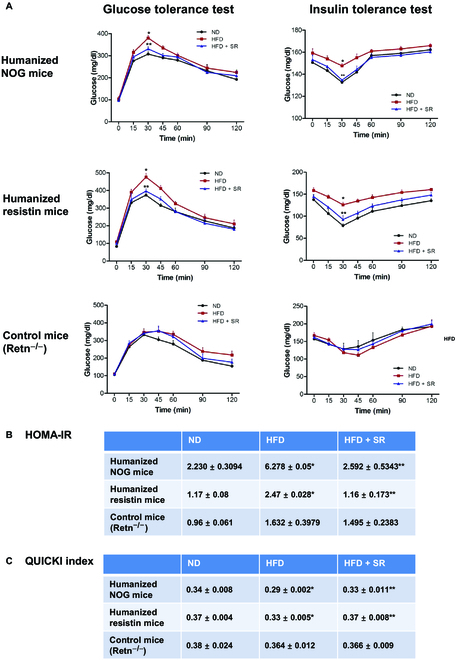
Glucose homeostasis in humanized NOG mice and humanized resistin mice. (A) Mice were fed with a normal diet or a high-fat diet; SR141716 was intraperitoneally injected for 8 weeks. Glucose tolerance test was measured at times 0, 15, 30, 45, 60, 90, and 120 min. Insulin tolerance test was measured at times 0, 15, 30, 45, 60, 90, and 120 min (*P < 0.05, ND versus HFD; **P < 0.05, HFD versus HFD + SR; *n* = 8 to 10) (B) Glucose (in milligrams per decaliter), insulin (in nanograms per milliliter) levels, HOMA-IR, and the QUICKI were determined (*P < 0.05, ND versus HFD; **P < 0.05, HFD versus HFD + SR; *n* = 8 to 10).

To assess the influence of adipose tissue inflammation induced by resistin-secreting CB1R-positive monocytes on glucose homeostasis, we performed glucose tolerance tests in both mouse models. The high-fat diet-fed mice showed impaired glucose tolerance compared to mice fed a normal diet, which was normalized by SR141716 treatment in both humanized NOG mice and humanized resistin mice (Fig. 4A). To evaluate insulin sensitivity, we calculated 2 parameters: the homeostatic model assessment for insulin resistance (HOMA-IR) and the quantitative insulin sensitivity check index (QUICKI). HOMA-IR was higher, whereas QUICKI was lower in the high-fat diet-fed mice than in the mice fed a normal diet, indicative of insulin resistance, and the index was normalized in SR141716-treated mice fed a high-fat diet (Fig. 4B and C). In the Retn^−/−^ mice, however, a high-fat diet with or without SR141716 did not significantly affect glucose tolerance or insulin sensitivity (Fig. 4A to C).

### High-fat diet-induced insulin resistance in humanized mouse models is accompanied by mitochondrial change caused by resistin

**Fig. 5.**
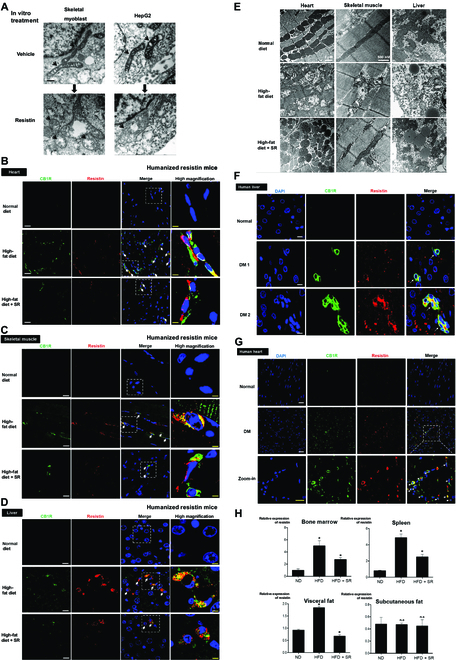
Insulin resistance facilitated by resistin may be associated with mitochondrial dysfunction induced by resistin. (A) Electron microscopic image of resistin treated human myoblast and hepatocellular carcinoma cells (HepG2) treated with resistin (50 ng/ml) for 3 h. It shows mitochondrial swelling, decreased matrix density, and distorted mitochondrial cristae in both cell types. Arrows indicate mitochondria. Scale bar, 100 nm. (B to D) The extent of the infiltrated cells secreting resistin was measured in target tissues such as the skeletal muscles, liver, and the heart. The high-fat diet-fed group showed increased number of resistin-positive cells in each organ, which was reversed by treatment with SR141716. Arrowheads indicate the cells that are positive for CB1R and resistin. Scare bars for immunofluorescence staining, 10 μm (for low power field) and 2.5 μm (for high power field). (E) Electron microscopy demonstrated that a high-fat diet changed the morphology of mitochondria into the swollen form, as seen in in vitro experiments. This change was reversed by treatment with SR141716. (F and G) Double immunofluorescence staining of human tissues such as the heart and liver between non-DM (normal) versus obese-DM (DM, diabetes mellitus) subject. Obese-DM subject showed a strong coexpression of infiltrated CB1R^+^ resistin^+^ monocytes (white arrows). Scare bars for immunofluorescence staining, 25 μm (for low power field) and 10 μm (for high power field). (H) Expression levels of resistin were measured in tissues of humanized resistin mouse models. Real-time PCR resistin in the bone marrow, spleen, visceral and subcutaneous fat of humanized resistin mice shows that high-fat diet group increased resistin expression that was reversed by SR141716 treatment (*P < 0.05, ND versus HFD, HFD versus HFD + SR; n.s., not significant; *n* = 4).

There are several possibilities explaining the underlying mechanism of insulin resistance. Among them, we focused on insulin resistance that might be induced by mitochondrial dysfunction. First, we evaluated the effect of resistin, an effector of the ECS–resistin axis, on the morphological change and function of mitochondria in human skeletal myoblast and HepG2 cells. Transmission electron microscopy showed that a low dose of resistin treatment (50 ng/ml) induced mitochondrial swelling and the distortion of mitochondrial cristae in both cell types (Fig. 5A). In addition, resistin treatment decreased oxygen consumption rate of mitochondria in a Seahorse XF analyzer, suggesting that resistin could induce mitochondrial dysfunction (Fig. S4A). Second, we analyzed the extent of the infiltrated cells secreting resistin in the target tissues such as the skeletal muscles, liver, and heart in humanized mouse models. The high-fat diet-fed group showed an increased number of resistin-positive cells in each organ, which was reversed by treatment with SR141716 (Fig. 5B to D and Fig. S4B to D). In parallel with the infiltration of resistin-positive cells, the morphology of mitochondria changed into the swollen form by a high-fat diet as seen in the previous in vitro experiments in electron microscopy (Fig. 5E and Fig. S8). This mitochondrial destructive change was prevented by treatment with SR141716.

Last, we examined existence of macrophages double-positive for CB1R and resistin in the tissues of obese diabetic patients. Strikingly, in double immunofluorescence staining of CB1R and resistin, we found a strong coexpression of CB1 and resistin in tissue-infiltrated macrophages in the liver and heart of obese diabetic patients but did not in the tissue of normal subjects (Fig. 5F and G). Next, we measured level of resistin in various tissues of humanized resistin mice. High-fat diet-fed mice showed an increased level of resistin in the bone marrow, spleen, and visceral fat, but not subcutaneous fat. High-fat diet led to increased level of resistin in tissues of humanized resistin mice, which was attenuated by administration of SR141716 (Fig. 5H).

These data suggest that a high-fat diet induces mitochondrial dysfunction through the ECS–resistin axis leading to insulin resistance.

## Discussion

### Tight association between the ECS and resistin

**Fig. 6.**
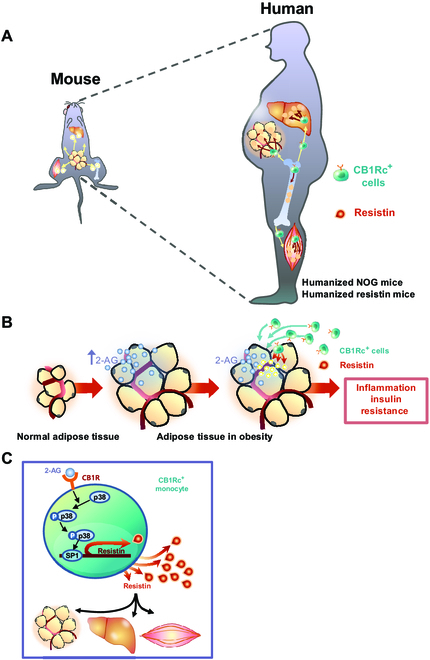
Schematic figure of CB1R-positive cells producing resistin. (A) CB1R-positive cells that secrete resistin may serve as mediators that elucidate the discrepancy between in mice and in humans in terms of the role of resistin in inducing insulin resistance. Mouse resistin is produced from adipose tissues; in contrast, human resistin is secreted by monocytes and macrophages. Owing to pathophysiological differences between mice and humans, we utilized animal models, such as humanized NOG mice and humanized resistin mice. (B) In both mouse models, a high-fat diet, which mimics the status of human obesity, increased the level of 2-AG in the visceral adipose tissue. Increased level of endocannabinoid ligands recruits the CB1R-positive cells into the target tissues, such as the adipose tissue, liver, and skeletal muscle. Mobilized CB1R-positive cells secrete resistin, which induces inflammation and insulin resistance in those tissues. (C) Resistin expression in CB1R-positive cells is regulated through the p38–Sp1 pathway.

Resistin, an adipokine that modulates insulin resistance, is linked to obesity and cardiovascular disease [7]. The association between resistin and insulin resistance in humans remains controversial because human resistin is secreted by monocytes and macrophages rather than adipocytes as in mice [12]. We focused on identifying a mediator that produces resistin in humans. In many aspects, the ECS and resistin share a common pathophysiology that includes obesity, inflammation, and glucose metabolism. Given the colocalization of CB1R and resistin in human atheroma, we assumed that there must be a CB1R-positive monocyte subset that produces resistin and infiltrates into the atheroma. We confirmed that circulating CB1R-postive cells secrete resistin and infiltrate into the target tissues such as visceral adipose tissue having high level of 2-AG, leading to inflammation and insulin resistance. By introducing the concept of circulating cells producing resistin, we can explain the different biology of resistin between rodents and humans to induce adipose tissue inflammation (Fig. 6). In rodents with small body size under a small loop, in situ adipocytes secrete resistin and induce adipose tissue inflammation. In contrast, in humans with big body size under complex collaboration of the ECS and resistin, ligands of the ECS accumulate in adipose tissue and attract CB1R-positive circulating monocytes that secrete resistin into adipose tissue, leading to inflammation. Many studies, including our own, have shown that conditions associated with obesity can lead to increased levels of 2-AG in target tissues, but not in the case of anandamide [17,18]. In addition, changes in 2-AG levels were observed specifically in the visceral adipose tissue, rather than in the subcutaneous adipose tissue. This observation correlates well with findings related to the infiltration of monocytes secreting resistin.

The CB1R is a member of the G protein-coupled receptors superfamily, and stimulation of CB1R leads to activation of mitogen-activated protein kinases [19]. Our findings indicate that 2-AG induced phosphorylation of p38, which was attenuated by CB1R antagonists in CB1R-positive cells. Moreover, increased resistin levels by 2-AG were attenuated by administration of a p38 inhibitor in CB1R-positive cells. Therefore, the ECS induces resistin through p38 activation in CB1R-positive cells.

### Adipose tissue inflammation via the endocannabinoid–resistin axis

The increased number of macrophages in adipose tissue is one of the hallmarks of obesity-linked inflammation [16]. When we analyzed the visceral adipose tissue in mice, the high-fat diet group showed an increased number of CLSs indicating macrophage infiltration. In obese mice, macrophages in adipose tissue undergo phenotypic change to the M1 type that has higher level of inflammatory cytokines such as TNF-α, IL-6, and IL-1β [16]. In our study, the majority of cells that infiltrated into the adipose tissue of obese mice was CB1R-positive cells secreting resistin. Both macrophage infiltration and induction of inflammatory genes were suppressed by CB1R blockers or under resistin knockout condition in vivo. Conversely, an endocannabinoid ligand, 2-AG, that accumulates in visceral adipose tissue under high-fat diets increased the expression of resistin and inflammatory cytokines in these cells. Furthermore, increased level of 2-AG recruited those cells into visceral adipose tissue. We observed 2-AG treatment increased resistin levels in human stromal vascular fraction cells that contain monocytes, but not in human white adipose tissue cell lines. This shows that 2-AG is exclusively effective to human monocytes and not adipocytes. These data suggest that the tight interplay of the ECS and resistin through resistin-secreting CB1R-positive cells plays an important role in linking obesity and adipose tissue inflammation. Thus, we can expect that new blockers of CB1R or resistin may be a promising solution to modulate adipose tissue inflammation associated with obesity and metabolic diseases.

### Atherosclerosis via the endocannabinoid–resistin axis

In humans, it is known that resistin induces inflammation not only in the adipose tissue but also in the arteries [10,20]. Until now, several mechanisms have been proposed to explain this phenomenon [21]. We previously reported that resistin is a causal factor of atherosclerosis by inducing adhesion molecules on endothelial cells and monocytes [10,20]. The prominent pathologic feature of atheroma is the infiltrated macrophages that are originally circulating monocytes in the blood [22]. Here, we found that resistin expression was colocalized with CB1R-positive cells in atheroma. Our study showed that among the monocytes in the peripheral blood, CB1R-positive cells express resistin, which provokes inflammation in diverse tissue types. On the basis of the findings of human atheroma and the presence of circulating CB1R-positive cells, we can assume that circulating CB1R-positive cells can infiltrate into the arteries and secrete resistin, resulting in the progression of atherosclerosis. Thus, the regulation of the interplay between the ECS and resistin could serve as a promising therapeutic strategy to prevent atherosclerosis.

### Insulin resistance and mitochondrial dysfunction induced by the endocannabinoid–resistin axis

There have been many studies demonstrating the underlying mechanism of insulin resistance [2,23–25]. One of them is related to the dysfunction of mitochondria. Impaired mitochondrial function contributes to decreased adenosine 5′-triphosphate production and β-oxidation and increased reactive oxygen species, which result in insulin resistance [26]. Our study demonstrated that in vitro resistin treatment to skeletal muscle cells or hepatocytes, 2 major target cells of insulin, induced mitochondrial swelling, decreased matrix density, and distorted mitochondrial cristae, indicating damaged mitochondria and mitochondrial dysfunction with the Seahorse analyzer assay. In addition, our animal models also demonstrated that high-fat diet-fed mice showed higher number of infiltrated cells producing resistin, which correlated with the mitochondrial changes. Those mice showed reduced insulin sensitivity. Importantly, CB1R antagonist treatment significantly reduced resistin-positive cell infiltration, the pathologic changes of mitochondria, and insulin resistance. We previously reported that resistin induces mitochondrial dysfunction, leading to obesity-induced metabolic diseases [27]. We showed that resistin binds to adenylyl cyclase-associated protein 1 (CAP1), leading to activation of mitochondrial-fission related protein, dynamin-related protein 1 (Drp1). Activated Drp1 is then recruited to mitochondria, assembling multimeric complexes and facilitates the scission of mitochondria. Abnormal mitochondrial fission by resistin leads to decrease in adenosine 5′-triphosphate production and mitochondrial dysfunction. Taken together, our data suggest that the endocannabinoid–resistin axis might regulate insulin resistance associated with mitochondrial dysfunction.

### New drug development based on the endocannabinoid–resistin axis

A CB1R antagonist, rimonabant, was developed as a modulator of obesity [28]. Randomized clinical trials showed a considerable reduction in body weight in subjects taking rimonabant [28–30]. In these studies, rimonabant also increased the level of high-density lipoprotein but decreased the level of triglyceride. In addition, patients treated with rimonabant exhibited increased levels of adiponectin. However, all these trials showed adverse events such as psychiatric disorders such as depression and anxiety, leading to withdrawal of the drug from the market. Recently, many pharmaceutical companies have attempted to develop a CB1R antagonist that does not cross the blood–brain barrier [31]. Therefore, new approach to overcoming this problem could be a great help to patients with cardiometabolic disease. Furthermore, we expect that our findings regarding the endocannabinoid–resistin axis could be a booster to develop more elegant ECS blockers.

In summary, our results provided the first evidence that the ECS and resistin are tightly associated, which could explain the key pathobiology of obesity, adipose tissue inflammation, mitochondria dysfunction, and insulin resistance. The ECS induces resistin expression in the CB1R-positive human peripheral monocytes that infiltrate into the target tissues associated with insulin resistance and inflammation. Therefore, these findings might provide new insight into the relationship between the ECS and resistin. Furthermore, the regulation of resistin via the CB1R might be a good therapeutic strategy for cardiovascular diseases by controlling obesity-related inflammation and insulin resistance.

## Materials and Methods

### Ethics statement

We obtained all human samples following written informed consent after receiving approval from the Institutional Review Board at Seoul National University Hospital (approval no. H-1001-021-306). All animal experiments were performed in accordance with the National Research Council “Guidelines for the Care and Use of Laboratory Animals”, after obtaining approval from the Institutional Animal Care and Use Committee at the Clinical Research Institute in Seoul National University Hospital.

### Cell isolation and culture

We used Ficoll-Paque PLUS (GE Healthcare) to isolate human PBMCs, following the manufacturer’s instructions. After washing the cells with phosphate-buffered saline, we stained the PBMCs with anti-CB1R (Abcam) and sorted them with a BD FACSAria II cell sorter for CB1R-positive cells. We determined the purity of CB1R-positive cells using flow cytometric analysis and resuspended the cells in endothelial cell basal media-2 (EBM-2) with 1% fetal bovine serum. To assess the impact of SR141716 (Santa Cruz Biotechnology), we pretreated the cells with or without 1 μM SR141716 in 10 μM 2-AG (Tocris Bioscience). For in vitro studies using recombinant human resistin, cells were serum starved for 3 h before resistin treatment. Following serum starvation, cells were treated with resistin (50 ng/ml; PeproTech, USA) for period indicated in figure legends.

### RNA isolation and polymerase chain reaction

We extracted total RNA using TRIzol Reagent (Invitrogen), adhering to the manufacturer’s instructions. We used 1 μg of total RNA for reverse transcription and amplified it using TaKaRa Ex-Taq. Real-time polymerase chain reaction (PCR) was conducted with SYBR Green Mix (Applied Biosystems) using an ABI prism 7500 (Applied Biosystems) under the following cycling conditions: 50 °C for 2 min, 95 °C for 10 min, 95 °C for 15 s, and 60 °C for 1 min for 40 cycles. The primers were as follows: human resistin, 5′-ctgtctcctcctcctccctg-3′ (forward) and 5′-caggccaatgctgcttattg-3′ (reverse); glyceraldehyde-3-phosphate dehydrogenase (GAPDH), 5′-gagtcaacggatttggtcgt-3′ (forward) and 5′-gacaagcttcccgttctcag-3′ (reverse).

### Western blot assay

We preincubated cells with either 1 μM SR141716 or 10 μM AM251 before treating them with 10 μM 2-AG. Cells were lysed with lysis buffers containing 50 mM tris (pH 7.2), 250 mM NaCl, 1% NP40, 0.05% SDS, 2 mM EDTA, 0.5% deoxycholic acid, 10 mM β-glycerol phosphate, 100 mM NaF, 1 mM orthovanadate, and protease inhibitor cocktail (Roche), and proteins were separated by SDS-polyacrylamide electrophoresis gel. Primary antibodies against human resistin (Santa Cruz Biotechnology), phospho-p38, p38, phospho-c-Jun N-terminal kinase (JNK), JNK, phospho-extracellular signal-regulated kinase (ERK), ERK (Cell Signaling Technology), and β-actin (Sigma-Aldrich) were used.

### Chromatin immunoprecipitation assay

We carried out the chromatin immunoprecipitation (ChIP) assay using the ChIP assay kit (Upstate Biotechnology), following the manufacturer’s instructions. We used sonicated lysate as an input control, and the rest of the lysate underwent immunoprecipitation with or without anti-Sp1 antibodies (Santa Cruz Biotechnology). The precipitated DNA fragments were analyzed by PCR with primers for the human resistin promoter using the 5′-ccacctcctgaccagtctct-3′ for forward primer and 5′-tgggctcagctaaccaaatc-3′ for reverse primer.

### Immunohistochemical and immunofluorescence staining

All animal experiments below were approved by the Seoul National University Institutional Animal Care and Use Committee and conducted in an Association for Assessment and Accreditation of Laboratory Animal Care (AAALAC)-approved animal laboratory. Bilateral mouse carotid arteries were exposed surgically under anesthesia. Perfusion fixation was performed by infusing 5% formaldehyde solution to both carotid arteries. On day of harvest, carotid arteries were removed, fixed, and prepared block for immunohistochemistry and immunofluorescence staining. We blocked sectioned and paraffin-embedded samples in 1% bovine serum albumin. We used primary antibodies against CB1R (Abcam), human resistin, and CD68 (Santa Cruz Biotechnology), followed by secondary antibodies. We obtained immunohistochemistry and fluorescence images using an upright and confocal microscope (Nikon ECLIPSE Ci, Carl Zeiss LSM710, Leica TCS SP8).

### Migration assay

We used Transwell Inserts (Becton Dickinson) to assess cell migration. We transferred cells suspended in EBM-2 medium to the insert and placed 2-AG in EBM-2 medium in the lower wells. After incubating at 37 °C and 5% CO_2_, we counted cells that migrated from the upper inserts to the lower wells.

### Mitochondrial respiration

The Seahorse XF24 extracellular flux analyzer (Agilent Technologies) was utilized to monitor mitochondrial oxygen consumption rates in a time-lapse approach [27]. Concisely, 3 × 10^4^ human skeletal myoblasts or HepG2 cells were cultivated in 1.5% gelatin-coated XF24 plates (Agilent Technologies) with each well holding a set [27]. The cells were nourished in complete growth medium overnight [27]. The succeeding day involved the addition of fresh medium [27]. Cells were thoroughly washed with phosphate-buffered saline twice before the day of the experiment and left to incubate in basal medium overnight [27]. On the day of the experiment, the starved medium was eliminated, and the cells were again washed twice with XF basal running medium (Agilent Technologies) enriched with 5.55 mM d-glucose and 1 mM sodium pyruvate and retained in 525 μl of running medium [27]. Each chamber received the specified drugs at concentrations indicated in the conditions section of methods [27]. The mitochondrial respiration was then tracked at 37 °C in 4 distinct, replicated wells [27]. Oxygen consumption rates and computed mitochondrial respiration parameters were assessed using Seahorse XF24 software and Seahorse XF Cell Mito Stress Test Summary Report.

### Transmission electron microscopy analysis

The cells underwent treatment with recombinant human resistin and subsequent harvesting via trypsinization. For transmission electron microscopy observation, cells and tissues were consolidated with 2.5% glutaraldehyde in 0.1 M phosphate buffer (pH 7.4) and subsequently with 2% osmium tetroxide in the same buffer. A series of ethanol was used to dehydrate the samples before embedding in resin. Sample sections were then prepared and inspected under a JEM-1400 microscope.

### Mice

Humanized NOG mice were created by irradiating NOG mice with 2.4 Gy, followed by a tail vein injection of 1 × 10^5^ human CD34-positive cells. Eight weeks later, FACS analysis confirmed the successful humanization of the mice. Humanized resistin mice, labeled CD68hR, were sourced from the University of Pennsylvania. Retn^−/−^ mice served as control mice. The mice received either standard feed or a high-fat diet (60% fat, research diets) over an 8-week period. To assess the impact of SR141716, the mice received an intraperitoneal injection of SR141716 (10 mg/kg).

### Glucose tolerance test and insulin resistance

After an overnight fasting period, a glucose tolerance test was administered. Blood glucose concentrations were assessed before and at 15, 30, 45, 60, 90, and 120 min following an intraperitoneal glucose injection (2 g/kg) utilizing an Accu-Chek. Blood glucose levels were captured at 0-, 15-, 30-, 45-, 60-, 90-, and 120-min intervals. Insulin levels were gauged using the UltraSensitive Mouse Insulin Enzyme-Linked Immunosorbent Assay Kit (ALPCO). The HOMA-IR and the QUICKI were computed as [G0 (mM) × I0 (μU/ml)/22.5] and 1/[log(I0) + log(G0)] respectively, where I0 represents fasting insulin (in microunits per milliliter) and G0 denotes fasting glucose (in millimolars) [32].

### 2-AG measurements

To measure 2-AG levels, tissues were homogenized in heptane/ethyl acetate (1:1, v/v) containing internal standards (1nmol of 2-AG-d8; Cayman). The resulting organic phase was then evaporated and reconstituted with 0.1% formic acid and 50% acetonitrile for further analysis via LC-MS/MS.

### Statistical analysis

All acquired data are represented as means ± SEM. The Mann–Whitney test and the Student’s *t* test were used where deemed suitable. The SPSS version 21.0 software (SPSS Inc., Chicago, IL) facilitated the analysis, and *P* values below 0.05 were considered statistically significant.

## Data Availability

The authors confirm that the data supporting the findings of this study are available within the article.
