# Supplementary material for: Resistin Regulates Inflammation and Insulin Resistance in Humans via the Endocannabinoid System
Source: Research (Wash D C). 2024 Apr 2;7:0326. doi: 10.34133/research.0326 (PMC11267475; doi:10.34133/research.0326)
Supplement: Supplementary 1 — Table S1 Figs. S1 to S9 [file research.0326.f1.docx]

**Supplementary Data**

**Supplementary Materials and Methods**

**Supplementary Table S1**

**Supplementary Figures S1-9 and Figure Legends**

**Supplementary Materials and Methods**

**Table S1. KEY RESOURCES TABLE**

| REAGENT or RESOURCE | SOURCE | IDENTIFIER |
| --- | --- | --- |
| Antibodies | | |
| anti-CB1R | Abcam | Cat# ab186428 |
| anti-Resistin | Santa Cruz Biotechnology | Cat# sc-15401 |
| anti-beta Actin | Sigma-Aldrich | Cat# A3854 |
| anti-pp38 | Cell Signaling Technology | Cat# 9211 |
| anti-p38 | Cell Signaling Technology | Cat# 9212 |
| anti-pJNK | Cell Signaling Technology | Cat# 9251 |
| anti-JNK | Cell Signaling Technology | Cat# 9252 |
| anti-pERK | Cell Signaling Technology | Cat# 9106 |
| anti-ERK | Cell Signaling Technology | Cat# 9102 |
| anti-Sp1 | Santa Cruz Biotechnology | Cat# sc-420 |
| anti-cd68 | Santa Cruz Biotechnology | Cat# sc-17832 |
| anti-cd14 | Santa Cruz Biotechnology | Cat# sc-1182 |
| anti-cd16 | Santa Cruz Biotechnology | Cat# sc-20052 |
| Goat anti-rabbit IgG-HRP | Santa Cruz Biotechnology | Cat# sc-2004 |
| Goat anti-mouse IgG-HRP | Santa Cruz Biotechnology | Cat# sc-2005 |
| Donkey anti-goat IgG-HRP | Santa Cruz Biotechnology | Cat# sc-2020 |
| Alexa 488 anti-rabbit IgG | Thermo Fisher Scientific | Cat# A-21206 |
| Alexa 488 anti-mouse IgG | Thermo Fisher Scientific | Cat# A-21202 |
| Alexa 555 anti-rabbit IgG | Thermo Fisher Scientific | Cat# A-21428 |
| Alexa 555 anti-mouse IgG | Thermo Fisher Scientific | Cat# A-21422 |
| Alexa 633 anti-rabbit IgG | Thermo Fisher Scientific | Cat# A-21070 |
| Alexa 633 anti-mouse IgG | Thermo Fisher Scientific | Cat# A-21050 |
| Chemicals, Peptides, and Recombinant Proteins | | |
| SR141716 | Santa Cruz Biotechnology | Cat# sc-205491 |
| 2-AG | Tocris Bioscience | Cat# 1298 |
| AM251 | Tocris Bioscience | Cat# 1117 |
| AM630 | Tocris Bioscience | Cat# 1120 |
| Recombinant human resistin | Peprotech | Cat# 450-19 |
| Critical Commercial Assays | | |
| Chromatin Immunoprecipitation assay kit | Upstate | Cat# 17-295 |
| Mouse Ultrasensitive Insulin ELISA | ALPCO | Cat# 80-INSMSU-E01 |
| Experimental Models: Cell Lines | | |
| Human skeletal muscle myoblasts | Lonza Inc | Cat# CC-2580 |
| Human: HepG2 | KTCC | Cat# HB-8065 |
| Oligonucleotides | | |
| Primers for resistin  Forward: ctgtctcctcctcctg  Reverse: caggccaatgctgcttattg | This paper | N/A |
| Primers for GAPDH  Forward: gagtcaacggatttggtcgt  Reverse: gacaagcttcccgttctcag | This paper | N/A |
| Primers for TNFα  Forward: tcagccgatttgctatctca  Reverse: cggactccgcaaagtctaag | This paper | N/A |
| Primers for IL-1β  Forward: ttgacggaccccaaaagatg  Reverse: agaaggtgctcatgtcctca | This paper | N/A |
| Primers for IL-6  Forward: acacatgttctctgggaaat  Reverse: aagtgcatcatcgttgttca | This paper | N/A |

**Animal and human studies**

All human samples were obtained with written informed consent after the approval by the Institutional Review Board (IRB) of Seoul National University Hospital. All animal experiments were performed after receiving approval from the Institutional Animal Care and Use Committee (IACUC) of Clinical Research Institute in Seoul National University Hospital and complied with the National Research Council (NRC) ‘Guidelines for the Care and Use of Laboratory Animals’.

**Cell isolation and culture**

Human peripheral blood mononuclear cells (PBMCs) were isolated by Ficoll-Paque PLUS (GE healthcare) according to the manufacturer’s instructions and were washed with phosphate buffered saline (PBS). For cannabinoid 1 receptor (CB1R)-positive cells, PBMCs were stained with anti-CB1R (Abcam) and sorted on a BD FACSAria II cell sorter. The purity of CB1R-positive cells was determined by flow cytometric analysis and cells were resuspended in EBM-2 with 1% FBS. To evaluate the effect of SR141716 (Santa Cruz Biotechnology), cells were pretreated with or without 1µM SR141716 in 10µM 2-AG (Tocris Bioscience).

**RNA isolation and PCR**

Total RNA was extracted using Trizol Reagent (Invitrogen) according to the manufacturer’s instruction. One microgram of total RNA was used for reverse transcription and was amplified by TaKaRa Ex-Taq. Real-time PCR was performed with SYBR Green mix (Applied Biosystems) using an ABI prism 7500 (Applied Biosystems). The cyclin conditions consisted of 50˚C for 2min, 95˚C for 10min, and 95˚C 15s, 60˚C for 1min for 40 cycles. The primers were as follows: forward 5’-ctgtctcctcctcctccctg-3’, reverse 5’-caggccaatgctgcttattg-3’ for human resistin and forward 5’-gagtcaacggatttggtcgt-3’, reverse 5’-gacaagcttcccgttctcag -3’ for GAPDH.

**Western blot assay**

Cells were preincubated with 1µM SR141716 or 10µM AM251 and then treated with 10µM 2-AG. Cells were lysed with lysis buffers containing 50mM Tris (pH 7.2), 250 mM NaCl, 1% NP40, 0.05% Sodium Dodecyl sulfate (SDS), 2mM Ethylenediaminetetraacetic acid (EDTA), 0.5% Deoxycholic acid, 10mM b-glycerol phosphate, 100mM NaF, 1mM Orthovanadate and Protease inhibitor cocktail (Roche) and proteins were separated by SDS-polyacrylamide electrophoresis gel. Primary antibodies against human resistin (Santa Cruz Biotechnology), phospho-p38, p38, phospho-JNK, JNK, phospho-ERK, ERK (Cell Signaling Technology) and b-actin (Sigma-Aldrich) were used.

**Chromatin immunoprecipitation (ChIP) assay**

ChIP assay was performed using the ChIP assay kit (Upstate Biotechnology) following the manufacturer’s instructions. The sonicated lysate was used as an input control, and the remaining lysate was for immunoprecipitation with or without anti-Sp1 antibodies (Santa Cruz Biotechnology). The precipitated DNA fragments were analyzed by PCR with primers for the human resistin promoter using the forward primer 5’-ccacctcctgaccagtctct-3’ and the reverse primer 5’-tgggctcagctaaccaaatc-3’.

**Immunohistochemical and immunofluorescence staining**

The paraffin-embedded samples were sectioned and blocked in 1% bovine serum albumin. The primary antibodies used were anti-CB1 receptor (Abcam), anti-human resistin and anti-CD68 (Santa Cruz Biotechnology) followed by Alexa Fluor 488, 555 and 633 secondary antibodies. The fluorescence image was obtained with a confocal microscope (Carl Zeiss LSM710).

**Migration assay**

The migration of cells was assayed using Transwell Inserts (Becton Dickinson). Cells suspended in EBM-2 medium were transferred to the insert and 2-AG in EBM-2 medium was placed in the lower wells. After incubation at 37˚C, 5% CO_2_ incubator, cells that migrated from the upper inserts to the lower wells were counted.

**Mitochondrial Respiration**

Time-lapse analysis of mitochondrial oxygen consumption rate was analyzed using the Seahorse XF24 extracellular flux analyzer (Agilent Technologies). Briefly, 3 x 10^4^ cells of human skeletal myoblasts or HepG2 cells were seeded in 1.5% gelatin coated XF24 plates (Agilent Technologies) per each well. Seeded cells were cultured overnight in complete growth media. Next day, fresh media was added. A day prior to experiment, cells were washed with PBS twice and incubated in the basal media for overnight. On day of experiment, starved media was removed, washed twice with XF basal running media (Agilent Technologies) supplemented with 5.55 mM D-Glucose and 1 mM sodium pyruvate and kept in 525 μl of running media. Each chamber was filled with desired drugs with a final concentration mentioned in conditions section of methods. Mitochondrial respiration was measured in a time-lapse manner at 37°C in 4 replicated, distinguished wells. Rates of oxygen consumption and calculated mitochondrial respiration parameters were analyzed using the Seahorse XF24 software and Seahorse XF Cell Mito Stress Test Summary Report.

**Transmission Electron Microscopy (TEM) analysis**

Cells were treated with recombinant human resistin and harvested by trypsinization. For observation under TEM, cells and tissues were fixed with 2.5% glutaraldehyde in 0.1M phosphate buffer (pH 7.4), followed by 2% osmium tetroxide in 0.1M phosphate buffer. The samples were dehydrated in a graded ethanol series and embedded in resin. The sample sections were cut and examined with JEM-1400.

**Mice**

For humanized NOG mice, NOG (NOD/Shi-scid, IL-2Rγnull) mice were irradiated with 2.4 Gy and 1x10^5^ human CD34 positive cells were transplanted through tail vein injection. After 8 weeks, mice were confirmed by FACS analysis as humanized NOG mice. Humanized resistin mice (designated CD68hR) were obtained from the University of Pennsylvania. Retn-/- (murine resistin knock out) mice were used as control mice. Mice were fed a normal chow or a high fat diet (60% fat, Research diets) for 8 weeks. To evaluate the effect of SR141716, mice were injected intraperitoneally with SR141716 (10mg/kg).

**GTT and insulin resistance**

Glucose tolerance test (GTT) was performed after an overnight fast. Blood glucose concentrations were measured before and 15, 30, 45, 60, 90, and 120 minutes after an i.p. injection of glucose (2g/kg) using an Accu-check. Blood glucose level was measured at 0, 15, 30, 45, 60, 90 and 120 minutes. Insulin levels were measured by UltraSensitive Mouse Insulin ELISA Kit (ALPCO). The homeostasis model assessment of insulin resistance index (HOMA-IR) was calculated as [G_0_ (mmol/l) x I_0_ (µU/ml)/22.5]. The quantitative insulin-sensitivity check index (QUICKI) was calculated as 1/[log(I_0_) + log(G_0_)]. I_0_ is fasting insulin (µU/ml) and G_0_ is fasting glucose (mmol/l).

**2-AG measurements**

In measuring the levels of 2-AG, the tissues were homogenized with Heptane/Ethyl acetate (1:1, v/v) containing internal standards (1nmol 2-AG-d8, Cayman). The organic phase was evaporated and reconstituted with 0.1% formic acid and 50% acetonitrile for analysis by liquid chromatography-tandem mass spectrometry (LC-MS/MS).

**Quantification and Statistical Analysis**

**Statistical Analysis**

All data are presented as means ± standard error of means (SEM). Mann-Whitney test and the student’s *t*-test were used when appropriate. SPSS version 21.0 (SPSS Inc., Chicago, IL) was used for the analysis and *P* values of *<0.05* were considered to be statistically significant.

**Supplementary Figure Legends**

**
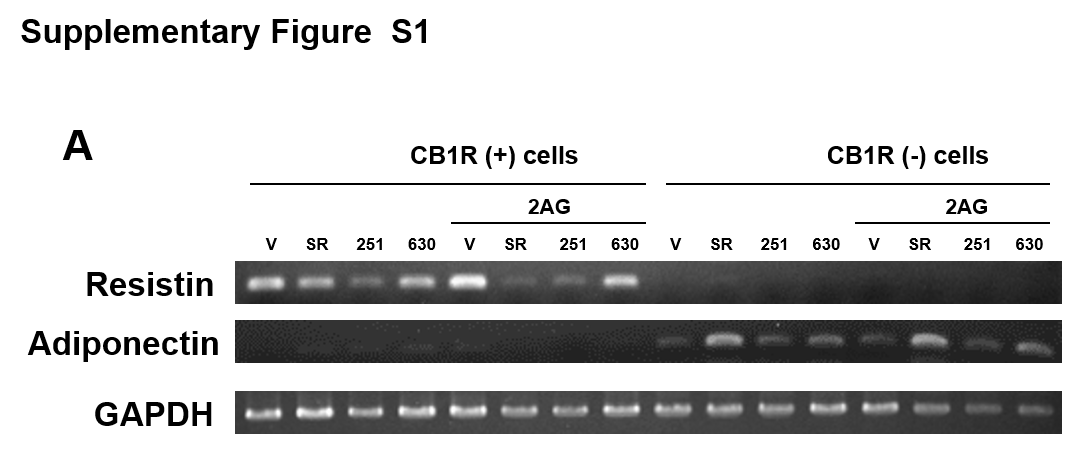
**

**Supplementary Figure S1.** Adiponectin expression in CB1R-positive and -negative fractions of PBMCs

(A) Resistin and adiponectin expression in sorted CB1R-positive or CB1R-negative cells, as determined by RT-PCR. Resistin and adiponectin expressions following treatment with 2-AG, with or without SR141716, AM251, and AM630 in CB1R-positive and -negative cells. Adiponectin expression was observed only in CB1R-negative fraction of cells, and was enhanced by treatment of SR141718. SR= SR141716; 251 = AM251; 630 = AM630.


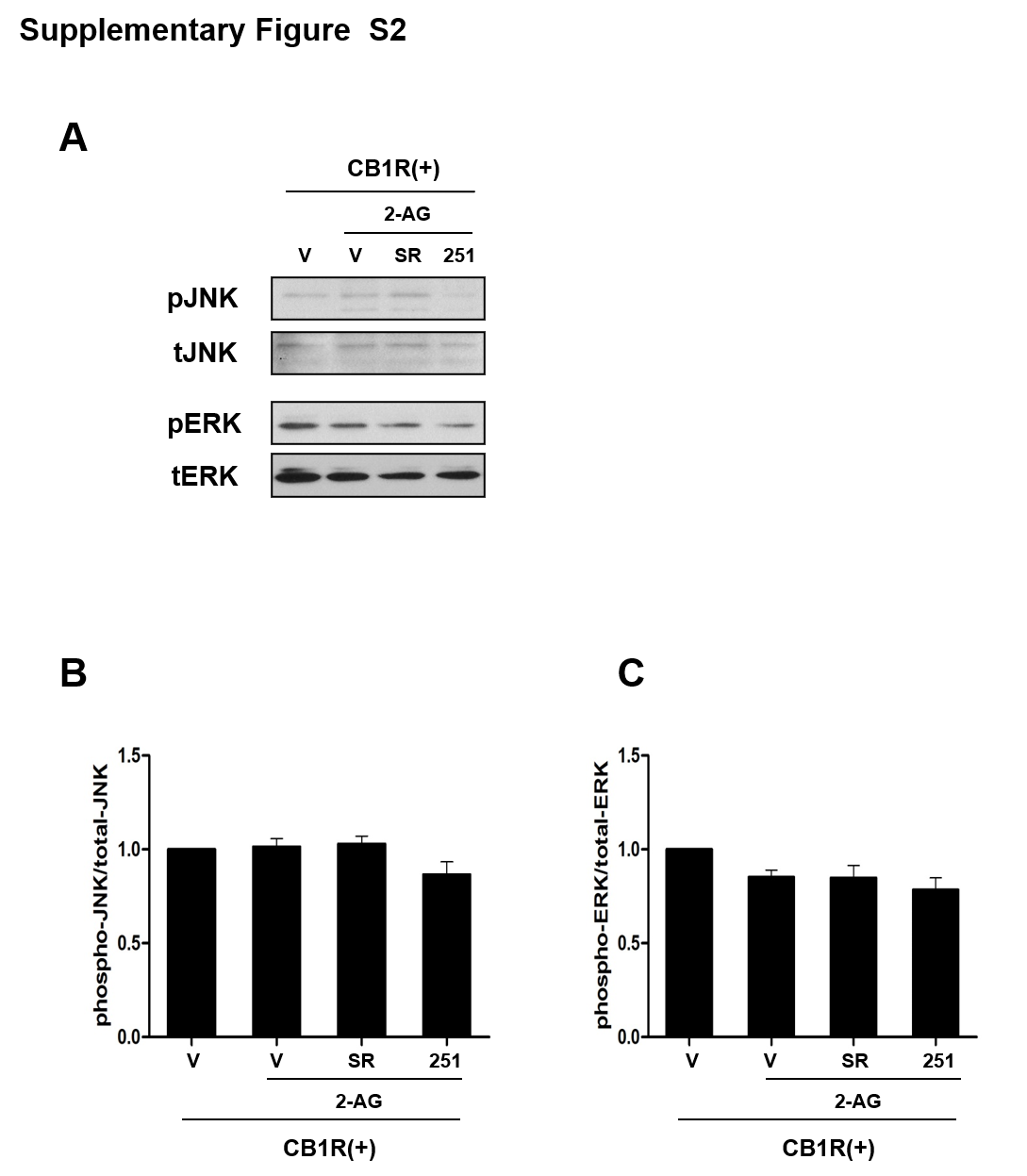


**Supplementary Figure S2.** Change in the phosphorylation of each MAPK induced by 2-AG and CB1R inhibitors

(A) Western blot analysis of JNK and ERK phosphorylation in CB1R-positive cells treated with 2-AG, SR141716, and AM251. pJNK = phospho-JNK; pERK = phospho-ERK; SR= SR141716; 251 = AM251. (B, C) Quantification of western blot assay. There was no significant change in JNK and ERK phosphorylation after treatment with 2-AG, SR141716, or AM251.


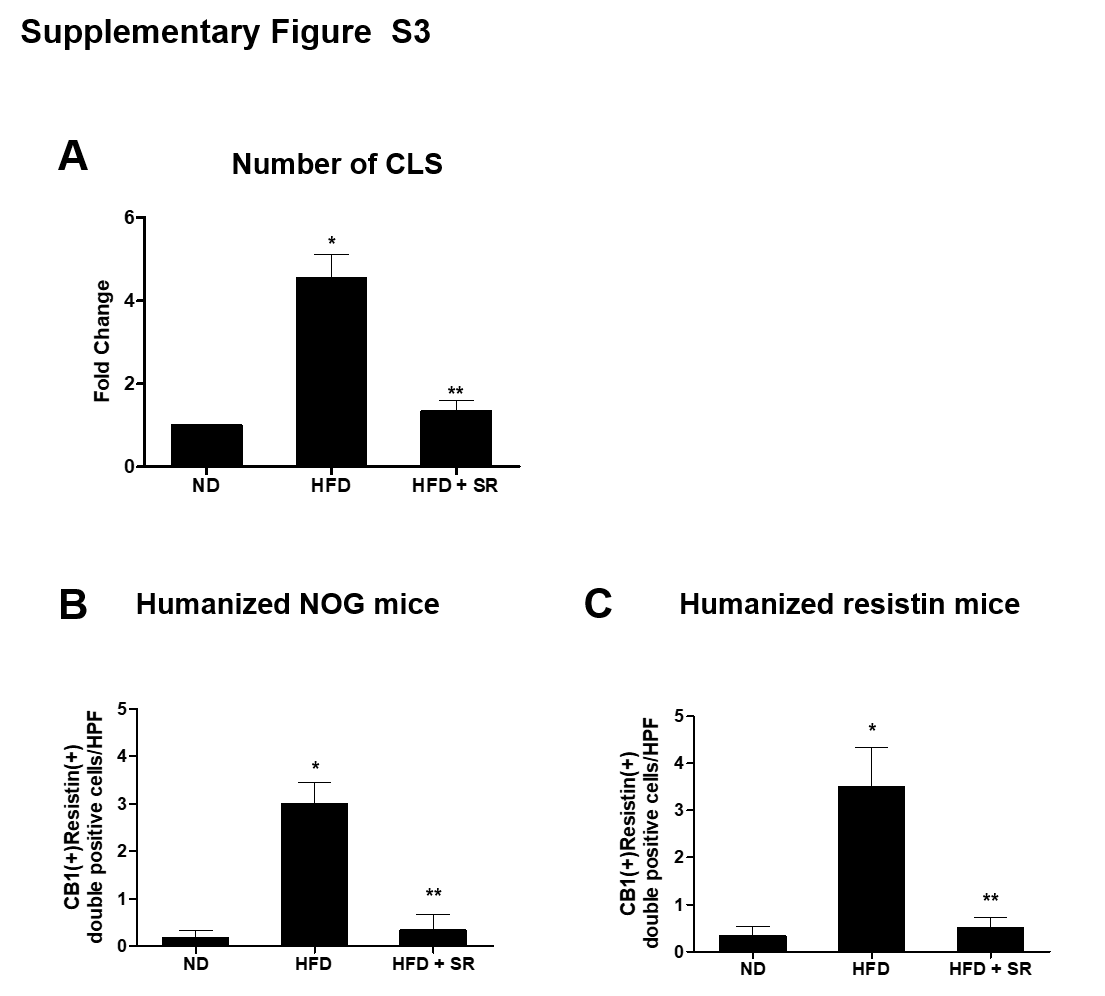


**Supplementary Figure S3.** Quantification data of each graph in *in vivo* experiments

(A) Quantification of the number of the crown-like structure (CLS) shown in the figure 2C. ND = normal chow diet; HFD = High fat diet; SR = SR141716. (* p < 0.05, ND vs. HFD; **p < 0.05, HFD vs. HFD + SR; n=6). (B) Quantification of the number of the double-positive cells for CB1 and resistin shown in the figure 2F. (* p < 0.05, ND vs. HFD; **p < 0.05, HFD vs. HFD + SR; n=6). (C) Quantification of the number of the double-positive cells for CB1 and resistin shown in the figure 2K. (* p < 0.05, ND vs. HFD; **p < 0.05, HFD vs. HFD + SR; n=5).


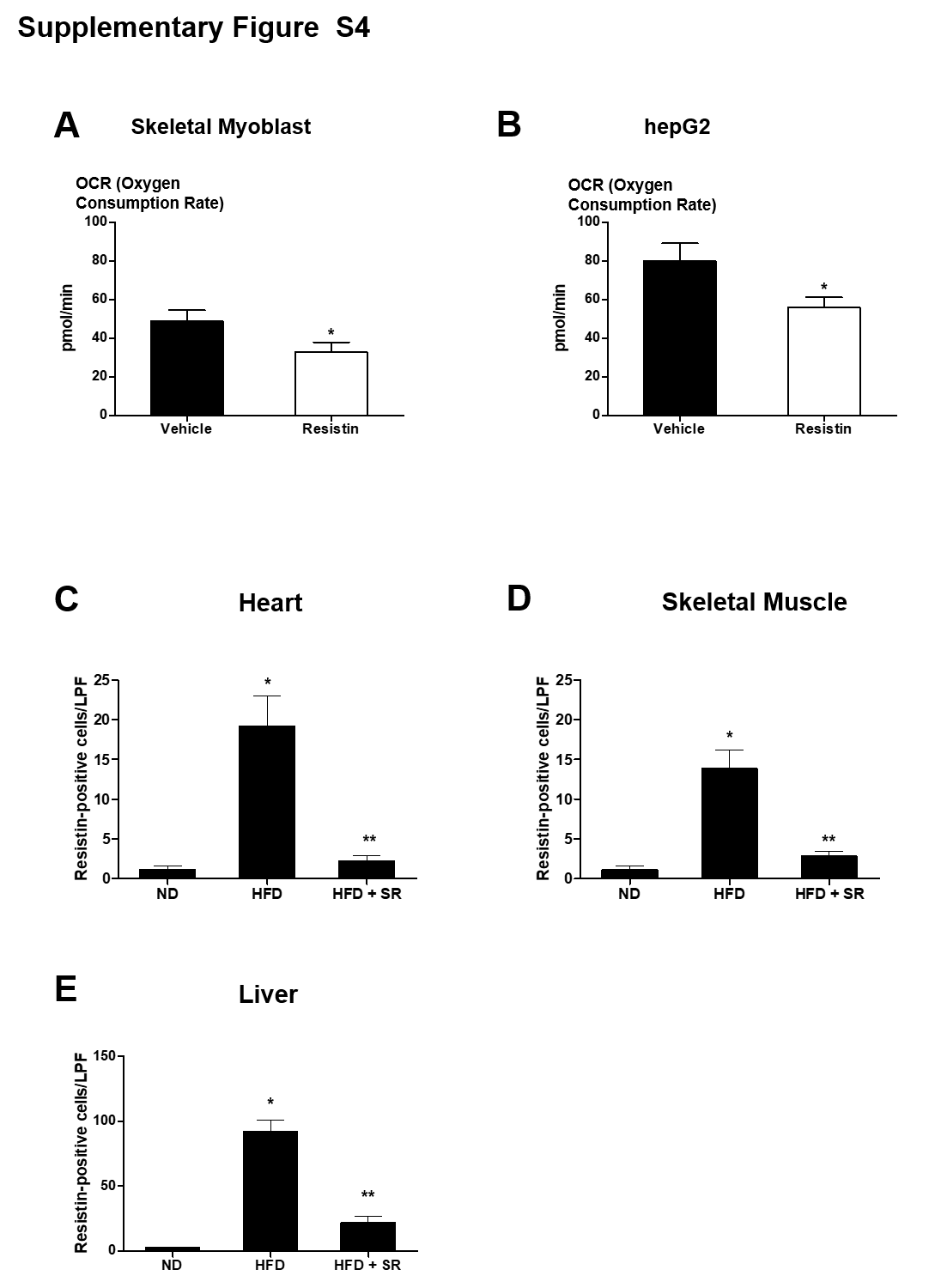


**Supplementary Figure S4.** The change of mitochondrial morphology and function after resistin treatment

(A, B) The results of oxygen consumption rate (OCR, ATP-linked) in a Seahorse XF analyzer indicates that resistin treatment decreased mitochondrial function in human skeletal myoblast and HepG2 cells. (* p < 0.05, vehicle vs. resistin treatment; n=6). (C-E) Quantification of the number of the resistin-positive cells shown in the figure 6B-D. ND = normal chow diet; HFD = High fat diet; SR = SR141716. (* p < 0.05, ND vs. HFD; **p < 0.05, HFD vs. HFD + SR; n=6).

**Supplementary Figure S5**


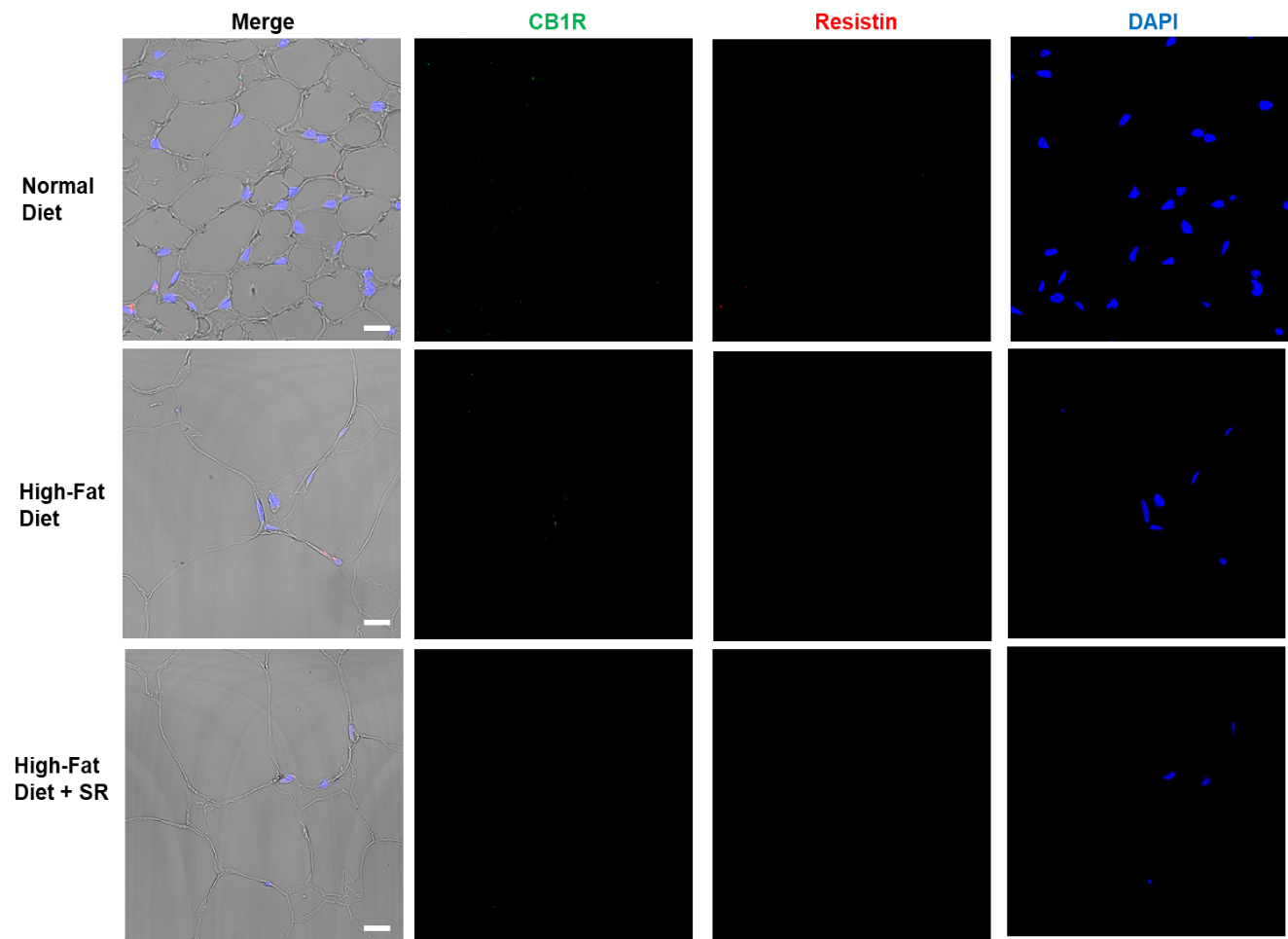


**Supplementary Figure S5.** Immunofluorescence staining of subcutaneous adipose tissue of humanized resistin mice fed with a normal diet, and a high-fat diet with or without CB1R antagonist, SR. CB1R and resistin expression were absent in subcutaneous adipose tissue. Scale bar: 20μM.

**Supplementary Figure S6**


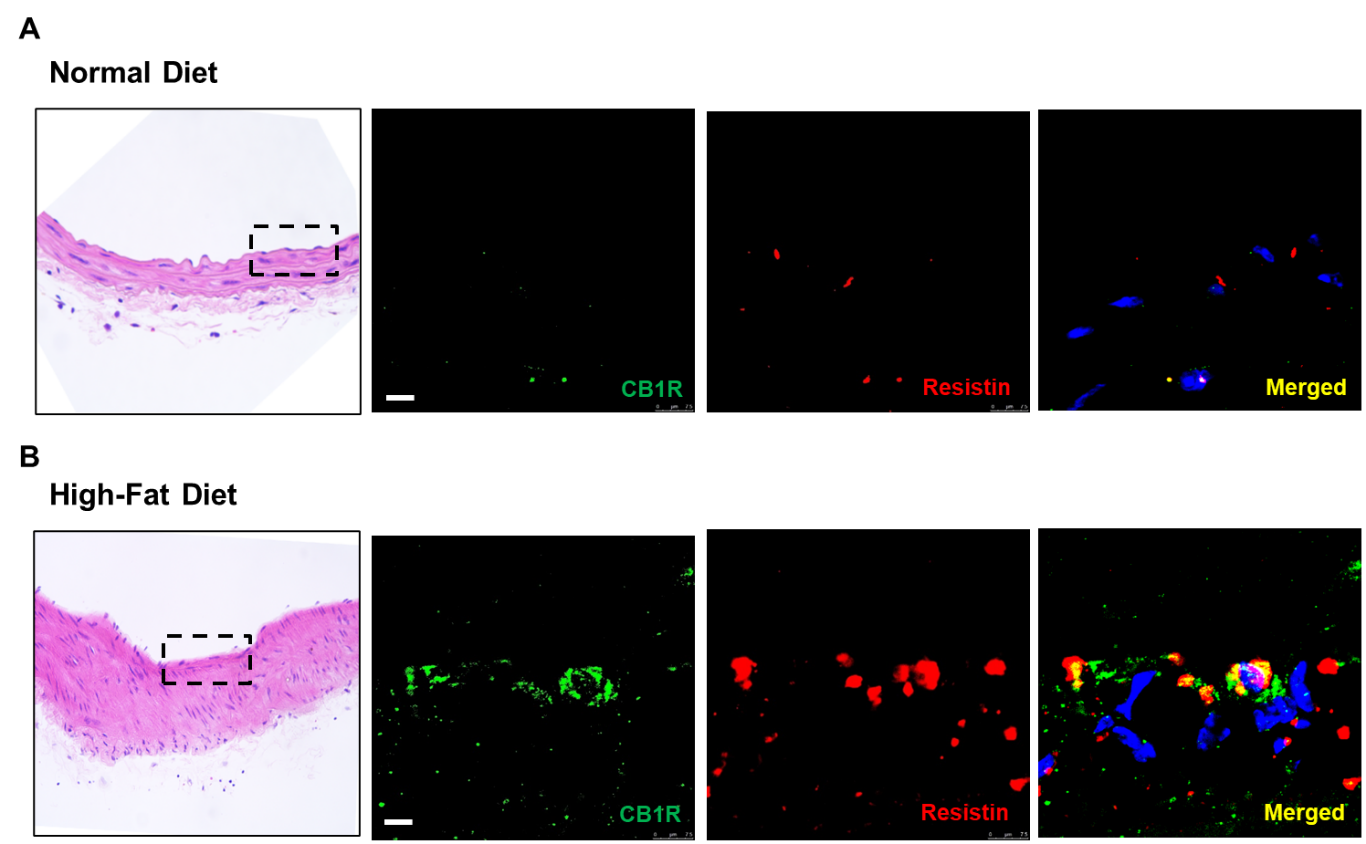


**Supplementary Figure S6.** Immunofluorescence staining of carotid artery of humanized resistin mice fed with a normal diet, and a high-fat diet. Fluorescence image of carotid artery of (A) normal-diet and (B) high-fat diet fed hRETN mice. CB1R and resistin are co-localized only in high-fat diet fed hRETN mice of carotid artery. Scale bar: 10μM.

**Supplementary Figure S7**


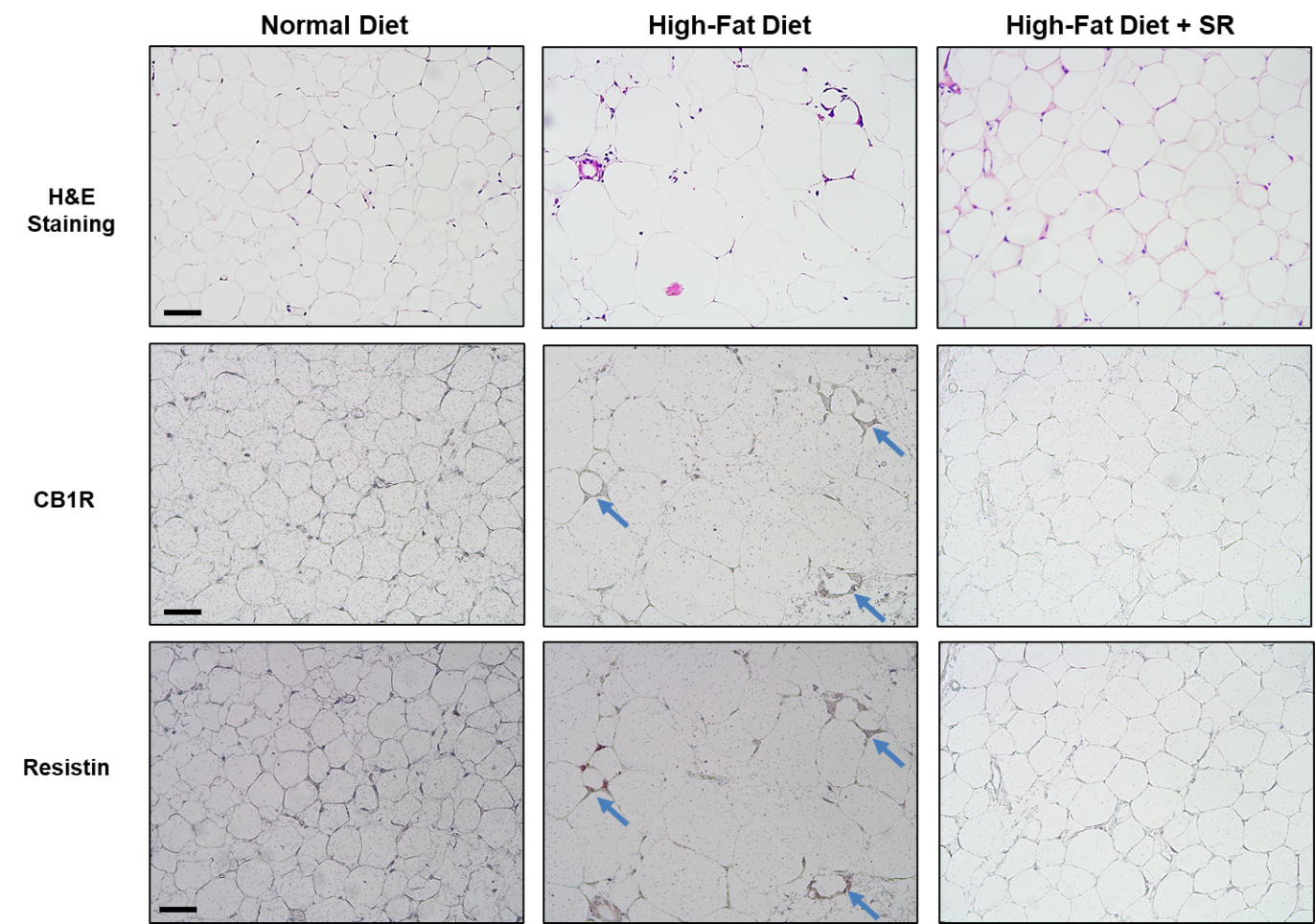


**Supplementary Figure S7.** Immunohistochemistry staining of visceral adipose tissue of humanized resistin mice merged with phase-contrast image. Paraffin-embedded mouse visceral adipose tissue blocks were adjacent sectioned and stained with anti-CB1R and anti-resistin antibody. CB1R and resistin positive cells are observed in same cell and equivalent region pointed out by solid blue arrow. Scale bar: 20μM.

**Supplementary Figure S8**


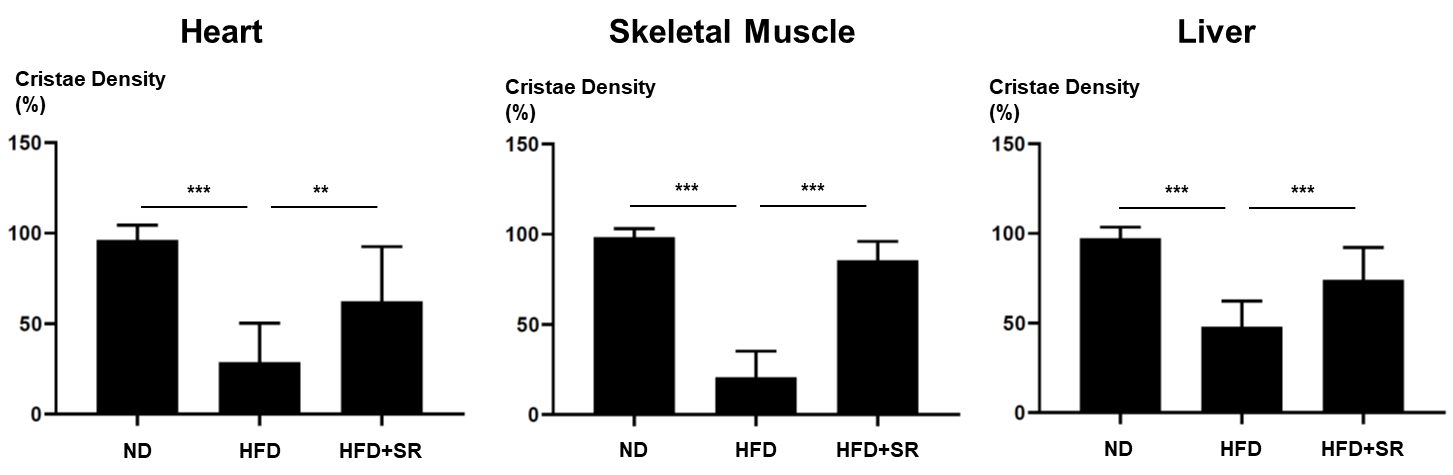


**Supplementary Figure S8.** Quantification of mitochondrial damage grade from electron microscopy images (Figure 5E). **p<0.05, ***p<0.005, > 50 mitochondria per group were quantified.

**Supplementary Figure S9**


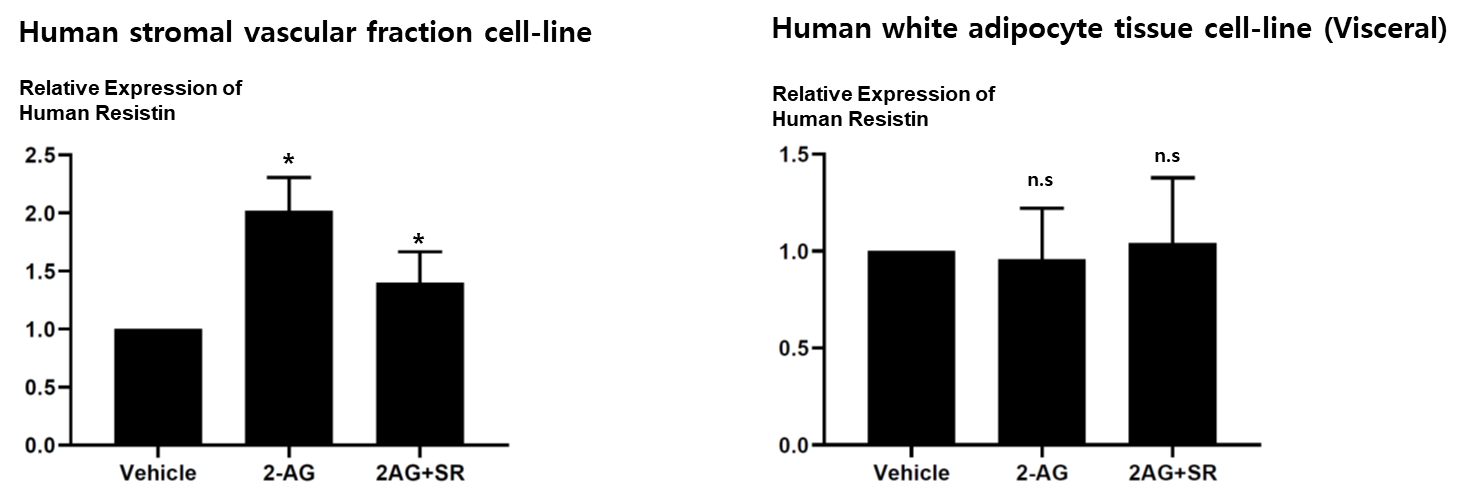


**Supplementary Figure S9.** Gene expression of human and mouse resistin in adipose tissue-derived cell-line. 2-AG treatment increased resistin expression in human stromal vascular fraction but not in adipocytes. *p<0.05, n=5
